# Systematic Screening of *Drosophila* Deficiency Mutations for Embryonic Phenotypes and Orphan Receptor Ligands

**DOI:** 10.1371/journal.pone.0012288

**Published:** 2010-08-19

**Authors:** Ashley P. Wright, A. Nicole Fox, Karl G. Johnson, Kai Zinn

**Affiliations:** 1 Division of Biology, California Institute of Technology, Pasadena, California, United States of America; 2 Department of Biology and Program in Neuroscience, Pomona College, Claremont, California, United States of America; Columbia University, United States of America

## Abstract

This paper defines a collection of *Drosophila* deletion mutations (deficiencies) that can be systematically screened for embryonic phenotypes, orphan receptor ligands, and genes affecting protein localization. It reports the results of deficiency screens we have conducted that have revealed new axon guidance phenotypes in the central nervous system and neuromuscular system and permitted a quantitative assessment of the number of potential genes involved in regulating guidance of specific motor axon branches. Deficiency “kits” that cover the genome with a minimum number of lines have been established to facilitate gene mapping. These kits cannot be systematically analyzed for phenotypes, however, since embryos homozygous for many deficiencies in these kits fail to develop due to the loss of key gene products encoded within the deficiency. To create new kits that can be screened for phenotype, we have examined the development of the nervous system in embryos homozygous for more than 700 distinct deficiency mutations. A kit of ∼400 deficiency lines for which homozygotes have a recognizable nervous system and intact body walls encompasses >80% of the genome. Here we show examples of screens of this kit for orphan receptor ligands and neuronal antigen expression. It can also be used to find genes involved in expression, patterning, and subcellular localization of any protein that can be visualized by antibody staining. A subset kit of 233 deficiency lines, for which homozygotes develop relatively normally to late stage 16, covers ∼50% of the genome. We have screened it for axon guidance phenotypes, and we present examples of new phenotypes we have identified. The subset kit can be used to screen for phenotypes affecting all embryonic organs. In the future, these deficiency kits will allow *Drosophila* researchers to rapidly and efficiently execute genome-wide anatomical screens that require examination of individual embryos at high magnification.

## Introduction

Most of the major findings that have emerged from research on *Drosophila* were driven by the identification of mutations producing a chosen phenotype *via* unbiased forward genetic screens. The pioneering anatomical screen of Nusslein-Vollhard and Wieschaus examined cuticle patterns of unhatched embryos bearing lethal mutations induced by the chemical mutagen ethyl methanesulfonate (EMS) [1]. The characterization of the genes found in this screen defined many of the fundamental mechanisms that control development in both insects and vertebrates.

Many other groups have since performed anatomical EMS screens of embryos. In the 1990s, Corey Goodman's group used antibody staining of whole-mount embryos to identify genes required for central nervous system (CNS) and motor axon guidance [2], [3]. These screens identified many interesting genes, including *roundabout* (*robo*) and *slit*, which control axon guidance across the CNS midline [4], [5]. However, the necessity to screen thousands of point mutant lines meant that axonal phenotypes had to be detected by examination of embryos at low magnification under a dissecting microscope. Subtle phenotypes could not be found in this manner. It can also be difficult to identify the gene responsible for a phenotype discovered in an EMS screen. *P* element insertion screens allow easier identification of mutated genes, but *P* element mutations are usually not nulls, and on average have weaker embryonic phenotypes than EMS mutations in the same genes.

Screens of deletion mutations, called deficiencies (Dfs), each of which removes multiple genes, have also been used to find genes required for embryonic development. Because many gene products are maternally expressed and deposited in the egg, where they can contribute to early development, embryos homozygous for Dfs can sometimes develop to fairly late stages. *single-minded* (*sim*), a gene required for midline glial fate determination, was identified through a Df screen [6], [7]. Embryos were stained with an antibody that labeled axons, and one Df was identified for which homozygotes had a CNS that consisted of a single line of axons running the length of the embryo. This phenotype was found to be due to the absence of a single gene, *sim*.

Df screening has also been employed to identify closely linked genes that have redundant functions. *reaper* (*rpr*), *hid*, and *grim* are regulators of cell death in *Drosophila* that are encoded by linked genes. A deletion that removed *rpr*, *hid*, and *grim* was found to eliminate all cell death in the embryo, but single mutations affecting the individual genes do not produce this phenotype. Thus, these cell death genes could only have been discovered by examination of Df phenotypes [8]. Similarly, the closely linked *pyramus* and *thisbe* genes, which encode partially redundant fibroblast growth factor-related ligands, were found by identifying a region whose deletion causes a failure of mesoderm spreading [9], [10].

Our group had devised methods to screen Df collections for genes required for expression of orphan receptor ligands [11], [12]. We realized that the Bloomington stock center Df ‘kit’ that existed in 2002, when we began these experiments, was of limited utility for screens requiring dissection and analysis of embryos. We thus began a project to define new kits of publicly available Dfs that could be used for ligand and antigen expression screens, as well as for phenotypic screening. Here we describe these kits, and present the results of screens that we have conducted for genes involved in nervous system development. We have found a variety of central nervous system (CNS) and motor axon guidance phenotypes, some of which represent new phenotypic classes. The kits should accelerate the work of investigators examining development of other embryonic organs, because they will allow high-resolution anatomical screens to be conducted much more rapidly than has been possible in the past.

## Results

### Development of a deficiency kit to screen for orphan receptor ligand genes

We initially used Df screening to search for ligands for the receptor tyrosine phosphatase (RPTP) Lar. We fused the extracellular (XC) domain of Lar to human placental alkaline phosphatase (AP), which is a dimeric protein [13], and expressed the resulting fusion protein (Lar-AP) using a baculovirus vector. Lar-AP-containing cell supernatants were incubated with live-dissected late stage 16 *Drosophila* embryos, and binding was detected with secondary and tertiary antibodies. Lar-AP stains a complex pattern, including CNS axons, midline glia, and muscle attachment sites.

To identify genes required for Lar ligand expression, we crossed green fluorescent protein (GFP) balancer chromosomes into the 270 lines in the Bloomington Df kit as it existed in 2002. We dissected GFP-minus late stage 16 embryos and stained them with Lar-AP. We found a Df that lacked Lar-AP muscle attachment site staining, and used overlapping Dfs and insertion mutations to identify the heparan sulfate proteoglycan (HSPG) Syndecan as the Lar ligand encoded within this Df [11].

In the course of this work, we dissected, stained, and analyzed embryos homozygous for every Df in the Bloomington kit. We found that homozygotes for many of the Dfs failed to develop to late stage 16, and thus could not be screened for ligand expression. Failure to develop is often due to the loss of zygotic expression of a single key gene under the Df. It is thus possible to reduce the sizes of these ‘unscreenable’ regions using other publicly available overlapping Dfs, examining homozygotes for each Df and finding those that develop well enough to be screened. We also tried to replace Bloomington kit Dfs that were only mapped to cytological resolution with molecularly mapped Dfs. After completing many iterations of this process, involving the dissection and staining of embryos from more than 700 Df lines, we were able to define a new kit of 423 lines, which allows screening of 80–90% of the genome for Lar-AP staining, or staining with other reagents that recognize CNS axons.

In creating this kit, we had two goals that had to be balanced against each other. The first was to assemble a set of lines that would have the highest possible percentage of Dfs for which homozygotes developed relatively normally. The second was to cover the maximum possible percentage of the genome with a minimum number of Dfs. The present kit is a compromise, as it still has a substantial number of Dfs that cause major developmental defects. However, we have tried to reduce the regions that are only covered by Dfs of this kind to the minimum size possible, by iterative screening of Dfs covering smaller and smaller portions of the ‘problem regions’ (see notes in Table S1). In some cases, we have reached the limit of our ability to reduce the sizes of the problem regions using existing Dfs. In other cases, it might be possible to subdivide the Dfs further to obtain greater coverage by Dfs that confer more normal development. However, this would mean that the resulting kit would contain more lines and would consequently be more time-consuming to screen.

We classified phenotypes for late stage 16 embryos homozygous for Dfs in the kit as ‘mild’, ‘moderate’, ‘severe’, or ‘very severe’. Homozygotes for 394 Dfs, corresponding to 82% of the genome (233 mild+83 moderate+78 severe) have a recognizable pattern of CNS axons and exhibit clear Lar-AP staining, as well as staining with the CNS axon marker monoclonal antibody (mAb) BP102 [2]. The 25 very severe Dfs that remain in the kit, representing another ∼10% of the genome, are marginally screenable with Lar-AP and BP102 (Table S1). The remaining ∼8% of the genome cannot be examined at present using Df screening, because embryos homozygous for Dfs in these regions fail to develop any recognizable late embryonic structures and cannot be dissected. We expect that the mild, moderate, and severe Df lines should also be readily screenable for ligands expressed outside the CNS, although development of the desired tissue will need to be evaluated for each Df by double-staining with an appropriate antibody, as we did for the CNS with BP102.

**Figure 1:**
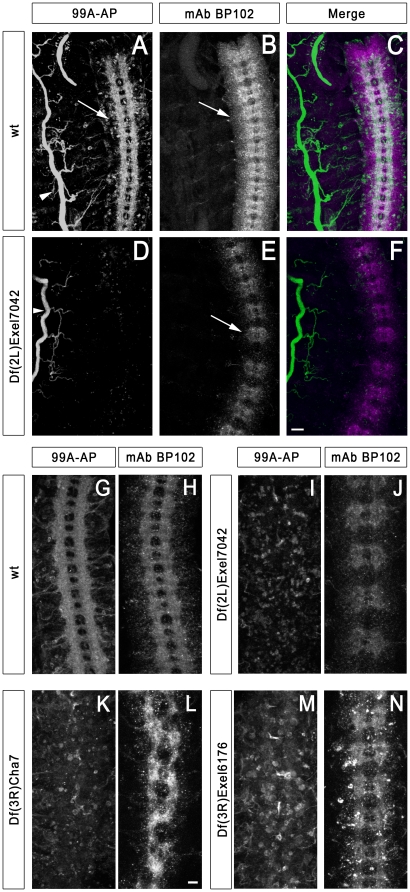
Deficiencies that reduce 99A-AP fusion protein staining of CNS axons. Confocal maximum intensity projections of late stage 16 live-dissected Drosophila embryos visualized by immunofluorescence (20× objective). Anterior is up. Panels A–F show the ventral nerve cord and one half of the body wall. Panels G–N show a portion of the ventral nerve cord. In Panels A–F scale bar equals 20 µm. In Panels G–N scale bar equals 10 µm. (A) 99A-AP fusion protein staining pattern in a wild-type embryo. The arrow indicates 99A-AP fusion protein binding to axons in the ventral nerve cord while the arrowhead indicates binding to tracheae. (B) mAb BP102 staining labels commissures and longitudinal connectives of the ventral nerve cord (arrow) in the same embryo. (C) Wild-type merged image (99A-AP, green; BP102, magenta). (D) 99A-AP fusion protein staining is absent from the ventral nerve cord in a Df(2L)Exel7042 embryo, while tracheal staining remains unaltered (arrowhead). (E) mAb BP102 staining in Df(2L)Exel7042 reveals that axons are still present in the ventral nerve cord (arrow). (F) Df(2L)Exel7042 merged image. (G and H) wild-type embryo stained with 99A-AP fusion protein and mAb BP102. (I and J) Df(2L)Exel7042 embryo stained with 99A-AP fusion protein and mAb BP102. (K and L) Df(3R)Cha7 embryo lacks 99A-AP fusion protein staining of CNS axons, but has axons as evidenced by BP102 staining (L). (M and N) Df(3R)Exel6176 embryo has reduced 99A-AP fusion protein binding to CNS axons, but has axons as evidenced by BP102 staining (N). Note that there appears to be more staining of cell bodies in the CNS than in (I) and (K).

We have now completed the screening of this new kit with four different RPTP-AP fusion proteins, corresponding to the XC domains of Lar, Ptp10D, Ptp69D, and Ptp99A. Here we show results from the Ptp99A ligand screening as an example of the method. In wild-type late stage 16 embryos, 99A-AP (visualized with Alexa 488-coupled anti-rabbit IgG) stains CNS axons, the tracheae, and the salivary glands (Figures 1A, G). CNS axons were simultaneously visualized by staining with BP102, followed by Alexa 568-conjugated anti-mouse IgG. BP102 stains most or all CNS axons, but only within the boundaries of the CNS. Motor axons lose BP102 staining after they leave the CNS. The BP102 pattern thus resembles a ladder, with two commissural tracts (anterior and posterior) crossing the midline in each segment, and two longitudinal tracts extending the length of the embryo (Figures 1B, H).

We found three Dfs for which homozygotes have reduced 99A-AP staining on CNS axons, but retain normal staining of the tracheae. Df(2L)Exel7042 and Df(3R)Cha7 both eliminate staining (Figures 1D, I, K). Df(3R)Exel6176 embryos have reduced axonal staining, and cell bodies within the CNS show more staining than in wild-type(Figure 1M). The lack of CNS axon staining in these Df homozygotes is not due to the absence of CNS axons, since BP102 staining reveals that axons are still present (Figures 1E, J, L, N). The decrease in 99A-AP axon staining is a robust phenotype that allows gene mapping, and we have used overlapping Dfs and insertion mutations to identify single genes under each of these three Dfs whose loss accounts for the reduction in staining. These genes are: *repo*, *gcm*, and *Cal1* (CG5148). *repo* and *gcm* encode transcription factors that control glial fate determination. The requirement for these genes demonstrates that a signal from glia to neurons is necessary for expression of the axonal Ptp99A ligand(s). We have confirmed this by killing glia and showing that this also eliminates axonal 99A-AP staining (data not shown). We do not yet understand the role of *Cal1*, which encodes a protein required for metaphase chromosome alignment in cultured cells [14], [15].

### A deficiency kit for screening for axon guidance phenotypes

In the course of our screen for genes required for RPTP ligand expression, we noted that homozygotes for 233 of the Dfs in our kit, corresponding to ∼50% of the genome, developed a normal or almost normal pattern of CNS axons as visualized by BP102, and also had relatively normal overall embryonic morphologies. 217 of these Dfs can be maintained over GFP balancers, so that homozygous embryos can be easily identified. We reasoned that these lines (classified as ‘mild’) could define a kit that would allow systematic screening for any embryonic phenotype that can be visualized by staining live-dissected embryos with antibodies (Table S2). We decided to search for motor axon guidance phenotypes by staining homozygotes for mild Dfs with mAb 1D4, which recognizes the cytoplasmic domain of the transmembrane form of Fasciclin II (FasII) [3]. In late stage 16 embryos, 1D4 stains all motor axons and three longitudinal axon bundles on each side of the CNS. We also stained a subset of the Dfs with moderate phenotypes with 1D4 in order to find new CNS phenotypes (see below).

Each abdominal hemisegment (A2–A7) of a Drosophila embryo contains about 36 motor neurons, which innervate 30 body wall muscle fibers in a stereotyped pattern. Motor axons leave the CNS within two nerve roots: the segmental nerve (SN) root and the intersegmental nerve (ISN) root. These nerve roots further divide into five major pathways, known as segmental nerve a (SNa), segmental nerve c (SNc), intersegmental nerve (ISN), intersegmental nerve b (ISNb) (also known as SNb), and intersegmental nerve d (ISNd) [16]. Two axons leave the CNS separately from the ISN and SN, and these connect to the axon of the peripheral lateral bipolar dendrite (LBD) neuron to form the transverse nerve (TN).

In the 1990s, 1D4 was employed for a large EMS screen of the autosomes [3], [17] for genes conferring embryonic motor axon guidance phenotypes. Because a point mutant screen requires the analysis of thousands of lines, these phenotypes were discovered through examination of whole-mount embryos at low power under a dissecting microscope. This allowed the identification of mutations conferring relatively severe ISN and ISNb phenotypes, which could be seen at low magnification. However, mutations affecting the other pathways, such as SNa and SNc, or mutations conferring more subtle ISN and ISNb phenotypes, could not be identified in this screen.

In our screen, we dissected live late stage 16 embryos homozygous for most of the balanceable mild Dfs (190 in total), fixed them after dissection, then stained with 1D4, followed by Alexa 488 anti-mouse secondary and rhodamine-phalloidin to visualize muscle structure and the CNS axon ladder. We examined these embryos under a compound microscope, using a 40× water-immersion objective. In this way, we could perform a detailed analysis of all of the motor pathways, and simultaneously visualize muscle structure.

To screen for motor or CNS axon guidance phenotypes, we typically examined five dissected late stage 16 embryos, or approximately 50 hemisegments. A Df was scored as phenotypically abnormal if multiple hemisegments in each embryo exhibited defects (>20% penetrance). 82 of the mild Dfs had no phenotypes with penetrances above this threshold. The remainder displayed axonal or muscle phenotypes, or both (Table S3).

### Examples of CNS phenotypes

**Figure 2:**
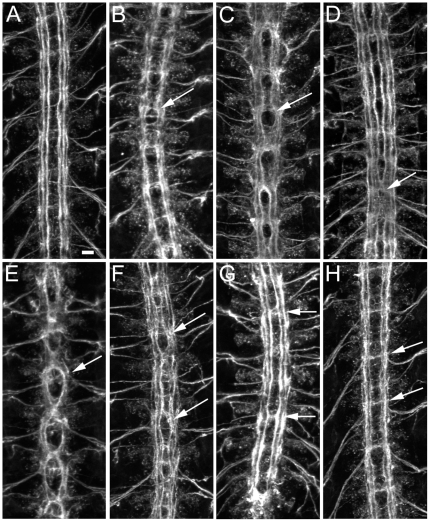
Examples of CNS phenotypes in deficiency homozygotes. (A–H) are confocal maximum intensity projections of mAb 1D4 immunofluorescence in live-dissected stage 16 embryos (20× objective). Anterior is up. Scale bar equals 10 µm. (A) wild-type embryonic nerve cord showing three parallel longitudinal bundles on either side of the midline and no FasII positive bundles crossing the midline. (B) Staining of FasII in *robo^1^* reveals a phenotype where axons repeatedly cross the midline in a circular fashion (arrow). This is a *robo^1^* phenotype of ‘average’ strength. Most published *robo* images are of unusually strong phenotypes. (C) Df(3R)Exel7310 embryonic nerve cord has a phenotype where axons appear to circle the midline (arrow). (D) *sad^1^*/Df(3R)Exel7310 has a phenotype in which FasII positive axons cross the midline. It is weaker than the phenotype in (C), however. (E) Df(1)BSC627 and (F) Df(2L)Exel8041 also have phenotypes where axons appear to circle the midline (arrows). Each of the three phenotypes has a unique overall appearance, however. (G) In Df(3L)HD1, FasII positive axons of the inner fascicle cross the midline inappropriately (arrows). (H) Df(3R)Exel6162 has a phenotype in which the nerve cord has a normal geometry, but one commissural bundle per segment is FasII positive (arrow).

In late stage 16 wild-type embryos, 1D4 stains three parallel axon bundles on each side of the midline (Figure 2A). At this stage, FasII is not seen on the commissures. Since most longitudinal axons cross the midline at some point in their trajectory, this means that FasII is restricted to the longitudinal portions of a subset of CNS axons. At earlier stages in development, FasII also labels some commissural pathways.

We found a variety of CNS phenotypes in our screens. Here we show some interesting examples of *robo*-like phenotypes, analyzed by 1D4 staining. *robo* encodes an immunoglobulin (Ig) domain superfamily member that is a neuronal receptor for the midline glial ligand Slit, which functions as a repellent in this context [4], [5]. In the absence of Robo, 1D4-positive axons have reduced sensitivity to the repulsive Slit signal. As a consequence, they cross the midline repeatedly, forming trajectories with a circular appearance (Figure 2B). We found several regions of the genome whose deletion results in phenotypes resembling that of *robo*, although all of the Df phenotypes are more severe than those of *robo* single mutants.

Df(3R)Exel7310, Df(1)BSC627, and Df(2L)Exel8041 embryos all have midline guidance defects in which FasII-positive axons appear to form circles around the midline (Figures 2C, E, F). Each Df has its own characteristic phenotype. Df(3R)Exel7310 deletes a candidate gene, *shadow* (*sad*), that has a published *robo*-like phenotype [18]. To determine whether the phenotype of this Df is due to loss of *sad*, we crossed the *sad^1^* point mutation to the Df. The resulting transheterozygous embryos also have *robo*-like phenotypes, although they are much weaker than those of Df/Df embryos (Figure 2D). Perhaps *sad^1^* is not a null mutation, or other genes under the Df contribute to the phenotype. The other two Dfs appear to define new genes whose absence causes axons to circle around the midline. We have used overlapping Dfs to map the gene responsible to a smaller region within Df(1)BSC627. The Df(1)BSC627 phenotype was mapped to the 7F7-8A2 interval using Df(1)BSC592 (has the phenotype when transheterozygous with Df(1)BSC627) and Df(1)Exel6241 (does not have the phenotype).

Figures 2G and H show Dfs for which 1D4-positive axons abnormally cross the midline without circling. In Df(3L)HD1 embryos (Figure 2G), axons in the inner 1D4 longitudinal pathway cross the midline. Several other Dfs also have this kind of phenotype. Df(3R)Exel6162 produces an interesting phenotype in which the CNS has a normal organization, but one of the commissural pathways is now 1D4-positive (Figure 2H). This phenotype could arise from abnormal guidance of a small subset of longitudinal 1D4 axons across the midline, or from ectopic expression of FasII on a commissural axon bundle.

### SNa guidance phenotypes

**Figure 3 pone-0012288-g003:**
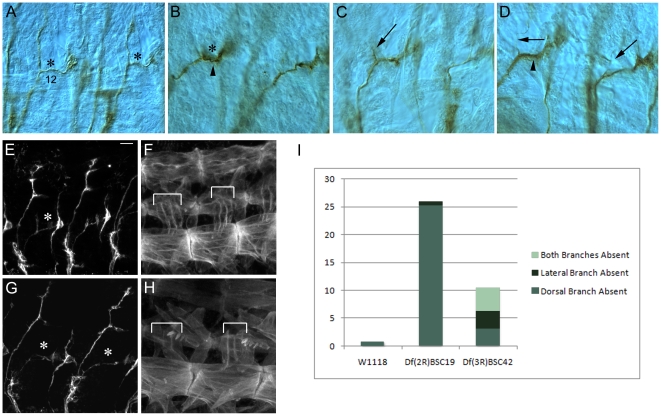
SNa defects in Df(2R)BSC19 and Df(3R)BSC42. (A–D) each show 2 abdominal hemisegments from late stage 16 embryos fixed and stained with mAb 1D4 using horseradish peroxidase immunohistochemistry (63× oil immersion objective and DIC optics). Anterior is to the left and dorsal is up. (A) The wild-type SNa has a characteristic bifurcated morphology where the dorsal branch innervates muscles 21–24 and the lateral branch innervates muscles 5 and 8. The bifurcation point is just dorsal to muscle 12 (labeled) and is indicated by an asterisk. (B) Df(2R)BSC19 abdominal hemisegments lack the dorsal branch of the SNa (asterisk) while the lateral branch appears thicker than wild-type (arrowhead). (C) Df(2R)BSC19 abdominal hemisegment where a short dorsal branch has extended but not reached the target muscles (arrow). (D) Df(2R)BSC19 abdominal hemisegments in which only one axon has extended dorsally (arrows). The lateral branch appears thicker in the left hemisegment (arrowhead). (E–H) Maximum intensity confocal projections of stage 16 live-dissected embryos double stained with 1D4 and rhodamine phalloidin (63× oil immersion objective). Anterior is to the left and dorsal is up. Scale bar equals 10um. (E–F) wild-type abdominal hemisegments showing a bifurcated SNa (asterisk). The target muscles of the dorsal branch of SNa (muscles 21–24) are indicated by brackets in (F). (G–H) Df(2R)BSC19 abdominal hemisegments showing the absence of the dorsal branch of SNa (asterisks) but the presence of target muscles 21–24 (brackets). (I) Chart showing quantification of various SNa defects in wild-type, Df(2R)BSC19 and Df(3R)BSC42. The number of abdominal hemisegments A3–A7 scored is as follows: wt  = 135, Df(2R)BSC19  = 155, Df(3R)BSC42  = 126.

The SNa exits the CNS in the SN nerve root. SNa has a characteristic bifurcated morphology in wild-type late stage embryos, with a dorsal (or anterior) branch and a lateral (or posterior) branch. The SNa bifurcation point is at the dorsal edge of muscle 12. The dorsal branch of the SNa innervates muscles 21–24, while the lateral branch innervates muscles 5 and 8 (Figures 3A, E). Each mild Df was screened for the presence of a bifurcated SNa pathway. Of the 190 lines screened, 20 had SNa bifurcation defects (Table 1).

**Table 1:** 

Phenotypic Class	Number of Dfs
SNa	20
ISNb	9
ISN	12
SNc	9
Muscle Defects	24
Multiple Pathways	9
Other	23
Total	106

The SNa pathway develops later than the other motor axon pathways, and is also fainter than the ISN or ISNb in 1D4-stained preparations. Live dissections can only be performed up to late stage 16, because older embryos fail to stick to the glass slides. We could not be sure that SNa defects seen in the live dissections were not due to developmental delays. Accordingly, we did not attempt to quantitatively score SNa bifurcation failures in live-dissected embryos. Instead, we generated early stage 17 whole-mount embryos stained with 1D4, using horseradish peroxidase (HRP) immunohistochemistry for detection, for a subset of the Dfs that had strong SNa defects. These were then dissected after clearing in glycerol and visualized with differential interference contrast (DIC) optics. Previously reported SNa phenotypes (e.g. *Ptp52F* [19]) usually involve the loss of either the dorsal or lateral branch with an approximately equal probability. Df(3R)BSC42 causes a typical SNa bifurcation defect in which the dorsal, lateral, or both branches are missing with nearly the same penetrances (3, 3, and 4% respectively in HRP-stained preparations; Figure 3I). However, Df(2R)BSC19 (56F12--14;57A4) produces a unique phenotype in which only the dorsal branch of SNa is absent or malformed (Figures 3B–D, G, and I). When we examined dissected whole-mount embryos from this line, we found that the dorsal branch of SNa was completely missing in 25% of hemisegments (n = 155) while the lateral branch is almost always present. Figure 3 shows three different Df(2R)BSC19 phenotypes affecting the dorsal branch: dorsal branch completely absent (Figure 3B), dorsal branch truncated (Figure 3C), and dorsal branch reduced to one axon (Figure 3D). Both SNa branches are present and have a normal thickness in >98% of hemisegments in wild-type embryos at the same stage (n = 135) (Figure 3I).

We examined whether the loss of the dorsal SNa branch in Df(2R)BSC19 embryos might be due to absence or malformation of its target muscles. Using phalloidin staining in live-dissected embryos, we determined that in 25% of abdominal hemisegments (n = 88) one or more of the dorsal branch target muscles (21–24) is not present, and this could account for the absence of the branch in these hemisegments. In live-dissected preparations, the SNa phenotype appears to be much stronger, so that 87% of hemisegments have a missing dorsal branch. In most of these (71% of total hemisegments), we find that the target muscles are in the proper place, yet SNa still fails to bifurcate (Figures 3G, H). The difference in the apparent strength of SNa phenotypes between live-dissected embryos stained fluorescently and dissected whole-mount embryos stained by HRP immunohistochemistry likely results primarily from the greater age of the whole-mount embryos. This was observed for other Dfs as well.

In many of the hemisegments in Df(2R)BSC19 embryos where the dorsal branch is missing or contains only one axon, there may be too many axons in the lateral branch, as this branch is abnormally thick (Figures 3B, D). In summary, the phenotype of Df(2R)BSC19 is likely to be a complex mixture of defects in axon guidance, defects in differentiation of lateral muscles, and developmental delays. We have narrowed down the responsible region of this Df to ∼6 genes, using Df(2R)BSC400 and Df(2R)Exel7163 (have the phenotype) and Df(2R)Exel7162 (does not have the phenotype). Whether all phenotypes are caused by the absence of a single gene remains to be determined.

### ISNb guidance phenotypes

**Figure 4 pone-0012288-g004:**
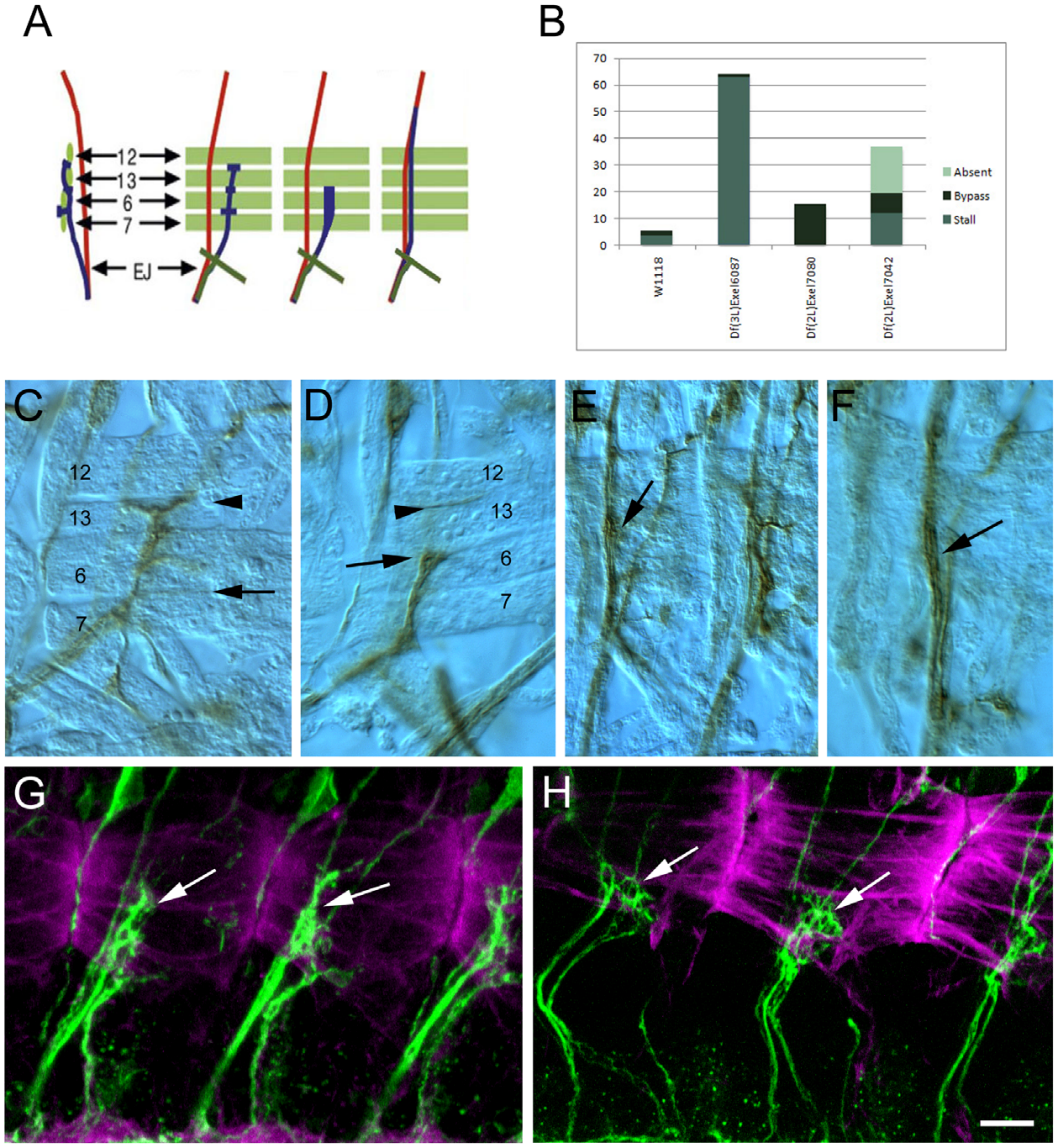
Examples of ISNb defects in deficiency homozygotes. (A) Schematic showing the axons of the of ISNb and their muscle targets in wild-type. Axons of the ISNb (blue) defasciculate from the ISN (red) at the exit junction (EJ) and enter the ventrolateral muscle field (green). The ISNd is in olive green. First panel is a side view with internal to the left. The second panel is a top view with anterior to the left and dorsal up. Two types of ISNb defects are illustrated in the third and fourth panels. The first defect is a stall of the ISNb where it does not project beyond the dorsal edge of muscle 6. The second defect is bypass, in which the axons of the ISNb do not enter the muscle field and grow dorsally past their normal muscle targets. Bypass phenotypes can represent a failure of ISNb axons to defasciculate from the ISN (fusion bypass), or successful defasciculation followed by a failure to enter the muscle field (parallel bypass). (B) Chart showing quantification of deficiencies with various types of ISNb defects. The number of abdominal hemisegments A3–A7 scored is as follows: wt  = 139, Df(3L)Exel6087  = 144, Df(2L)Exel7080  = 168, Df(2L)Exel7042  = 118. (C–F) each show abdominal hemisegments from late stage 16 embryos fixed and stained with mAb 1D4 using horseradish peroxidase immunohistochemistry (63× oil immersion objective and DIC optics). Anterior is to the left and dorsal is up. (C) ISNb in a wild-type embryo. The projection onto muscles 6 and 7 is indicated by an arrow. The projection onto muscles 12 and 13 is indicated by an arrowhead. The muscles are labeled with numbers. (D) ISNb in an embryo from Df(3L)Exel6087 showing a stall at the boundary between muscles 6 and 13 (arrow). Arrowhead indicates the normal ISNb termination point at the muscle 12/13 junction. The muscles are present and look wild-type and are labeled with numbers. (E–F) ISNbs in Df(2L)Exel7080 embryos showing bypass phenotypes (arrows). (G–H) are confocal maximum intensity projections from stage 16 live dissected embryos fluorescently stained with mAb 1D4 and rhodamine-phalloidin. These embryos are younger than in panels C–F because the loop phenotype in panel H is more evident at this stage. Scale bar equals 10 µm. (G) Three hemisegments of a wild-type embryo, showing ISNb (arrows) projecting normally into the muscle field. (H) Three hemisegments of a Df(2L)ED1317 embryo, where ISNb forms looping structures on the target muscles (arrows). This phenotype was not visible when embryos were fixed, dissected, and stained with the same antibody using HRP immunohistochemistry.

The ISNb contains the axons of the RP1, RP3, RP4, and RP5 neurons, among others. These axons cross the midline and exit the CNS in the ISN nerve root. They must defasciculate from the common ISN pathway at the ‘exit junction’ in order to enter the ventrolateral muscle (VLM) field. Once there, they innervate muscles 6, 7, 12, 13, 14, and 30 (Figures 4A, C). Of the 190 lines screened, 9 were found to have strong ISNb defects. These ISNb phenotypes include ‘bypass’, in which ISNb axons fail to separate from the ISN and grow past the VLMs, and ‘stall’, in which axons enter the VLM field but fail to reach the normal ISNb termination point at the ventral edge of muscle 12 (Figure 4A). We also found some structural abnormalities in the ISNb that could not be grouped into one of these classes.

Df(3L)Exel6087 produces a highly penetrant stall of the ISNb at the dorsal edge of muscle 6, which corresponds to the ventral edge of muscle 13. We quantified this phenotype in fixed-dissected embryos stained by HRP immunohistochemistry, and found that in 63% of hemisegments (n = 144) the ISNb fails to extend across muscle 13 (Figures 4B, D). Muscles 12 and 13 are present and appear normal both by phalloidin staining and DIC optics. Df(2L)Exel7080 causes a bypass phenotype in which 14% of hemisegments (n = 168) have no ISNb axons that grow into the VLM field (Figures 4B, E, F). We have identified *Mtp* (CG9342) as the single gene under this Df whose loss is responsible for the bypass phenotype (A. Cording and A.P.W., unpublished results).

Df(2L)ED1317 has an interesting structural phenotype in which ISNb axons form loops on muscles 6 and 7 (Figure 4H). When we examined Df(2L)ED1317 embryos stained as whole-mounts and visualized by HRP immunohistochemistry, we could not see this phenotype. It is likely that fine axonal branches such as those in the loops can be more readily seen in fluorescent preparations, because they can be damaged by the reactive oxygen species produced in the HRP reaction. Also, the aspect of the phenotype in which the axons failed to grow across muscle 13, which should have appeared as a stall in preparations stained by HRP immunohistochemistry, may have corrected itself later in development, so that it was no longer evident in the older embryos that were stained as whole-mounts. These results, together with observations we have made on other Dfs, show that some phenotypes that are readily seen in fluorescently stained embryos that are fixed after live dissection cannot be detected in dissected whole-mount embryos stained by HRP immunohistochemistry. Eight of the 9 regions whose deletion selectively affects ISNb guidance without visibly altering muscle development appear to define genes that have not been previously shown to affect motor axons. We examined the relationships between the Dfs and the locations of previously identified genes affecting the ISNb, to determine why we did not recover more Dfs spanning known ISNb guidance genes. We did find Dfs spanning one known gene, *sidestep* (*side*), but had classified these as affecting multiple pathways, since *side* mutants also have SNa and ISN defects [17]. For other known ISNb axon guidance genes with high-penetrance mutant phenotypes, the Df in our complete kit that spanned the gene was not within the subset of mild Dfs. For example, the *Lar* gene could not have been discovered using the mild Df kit, because the *screw* gene is embedded in one of its introns. Deletion of *screw* causes early developmental failure [20]. The only Df in our complete kit that removes *Lar* sequences also spans *screw*, and thus has a very severe phenotype.

### Requirement of the *glial cells missing* genes for ISNb guidance

**Figure 5:**
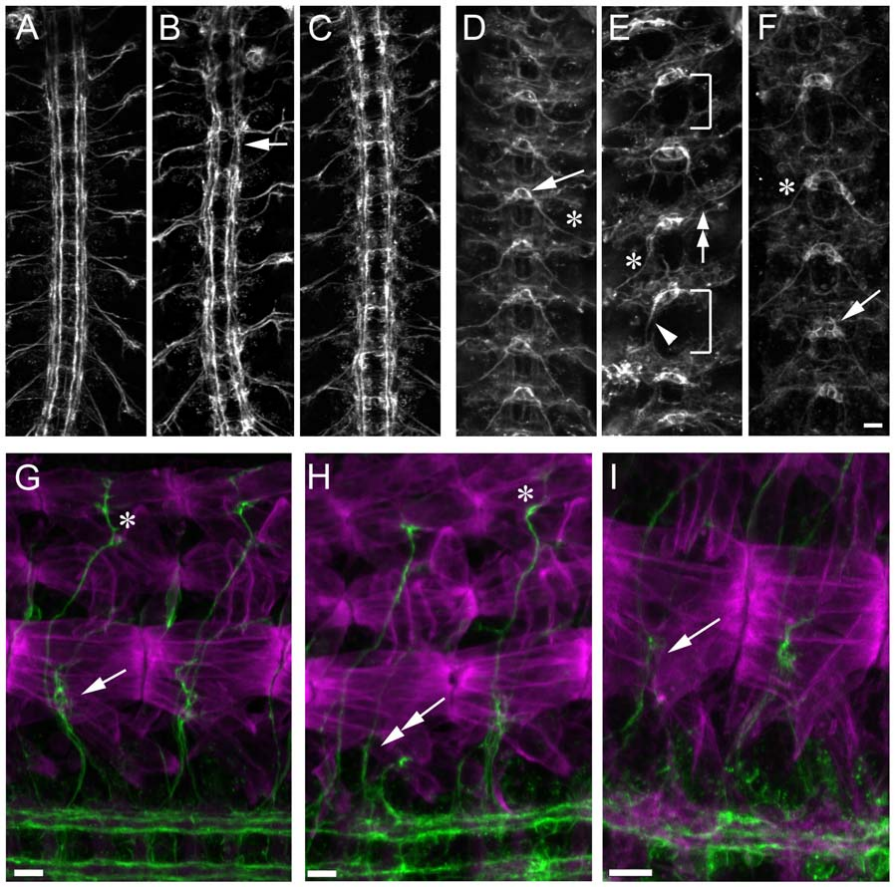
CNS and motor axon guidance phenotypes in mutants affecting glial development. (A–C) are confocal maximum intensity projections of the CNS in stage 16 live-dissected embryos stained with mAb 1D4 by immunofluorescence. Anterior is up. Scale bar in F equals 10 µm. (A) Ventral nerve cord of a wild-type embryo where mAb 1D4 labels 3 longitudinal bundles on either side of the midline. (B) Df(2L)Exel7042 embryo where breaks in the outer fascicle are evident in the longitudinal bundles (arrow). (C) *repo^3702^* embryo where ventral nerve cord appears disorganized. (D–F) are confocal maximum intensity projections of stage 16 live dissected embryonic CNS showing FasIII immunofluorescence. Scale bar equals 10 µm. (D) FasIII staining in a wild-type embryo reveals neurons of the RP cluster indicated by an arrow and their proximal projections into the periphery indicated by an asterisk. These axons cross the midline and then project posteriorly before leaving the nerve cord. (E) Df(2L)Exel7042 embryo where FasIII staining shows a very disorganized CNS. Axons sometimes project normally (asterisk), project posteriorly and never leave the CNS (arrowhead), or project anteriorly instead of posteriorly (double arrow). Some neurons never send out axons (brackets). (F) *repo^3702^* embryo where more cells than normal express FasIII (arrow). Axons occasionally project anteriorly instead of posteriorly, but the projections of most axons are normal (asterisk). (G–I) are confocal maximum intensity projections from live-dissected stage 16 embryos stained fluorescently with mAb 1D4 and rhodamine conjugated phalloidin. Anterior is to the left and dorsal is up. The scale bars are equal to 10 µm. (G) Two hemisegments of a wild-type embryo. Projection of the ISN is labeled with an asterisk. ISNb projections onto target muscles are indicated by an arrow. (H) Two hemisegments of Df(2L)Exel7042 embryo. The ISN projects normally (asterisk), but the ISNb is missing from the left hemisegment. The SN root is at an abnormal position (double arrow). See bar graph in Figure 4 for penetrance of the various ISNb defects in this deficiency line. (I) Two hemisegments from a *repo^3702^* embryo. In the left hemisegment, the ISNb stalls on muscle 6, and appears to be thinner than usual (arrow). Such phenotypes are rare in *repo* mutants.

Df(2L)Exel7042 has an ISNb phenotype that is a mixture of stall, bypass, and absence, in which there is no ISNb at all (12, 8, and 17% of hemisegments, respectively; n = 118) (Figure 4B). The CNS has a wavy 1D4 pattern with occasional breaks in the longitudinal tracts (Figure 5B). When we assessed the genes that are deleted by this molecularly mapped Df, two obvious candidates were found: *glial cells missing* (*gcm*) and *glial cells missing 2* (*gcm2*). *gcm* and *gcm*2 are transcription factors that are expressed in all glia except for midline glia and are required for glial cell fate determination [21], [22], [23]. In the absence of Gcm protein, some presumptive glial cells become neurons. When Gcm or Gcm2 is ectopically expressed in neurons, some of them become glia [21], [22], [24], [25]. *gcm* therefore acts as a molecular switch between the neuronal and glial cell fates. *gcm* and *gcm2* have largely overlapping expression patterns, with *gcm* being expressed at much higher levels than *gcm2*. In *gcm* mutants, a few glial cells remain, usually those in which *gcm2* expression is strongest. In the absence of both Gcm proteins, no glial cells are found [24], [25].

CNS axon guidance phenotypes have been previously observed in *gcm* mutants, including breaks in the longitudinal fascicles and abnormal trajectories of the pioneer axons of both the ISN and SN nerve roots [21], [22], [23], [26], [27]. Motor axon pathways usually develop normally in *gcm* mutants, although a published image of a *gcm* embryo has a missing ISNb in one hemisegment [27]. When we stained *gcm* null alleles with 1D4, we found that ISNb is affected, but with a much lower penetrance than in Df(2L)Exel7042 embryos, and the missing ISNb phenotype is almost never observed (data not shown). Thus, the deletion of both *gcm* and *gcm2* by Df(2L)Exel7042 could account for the severity of its ISNb phenotype. We also examined homozygotes for another Df which deletes both *gcm* and *gcm2*, Df(2L)200, as well as Df(2L)Exel7042/Df(2L)200 transheterozygotes. Homozygotes for a very small Df, Df(2L)ED684, which deletes only 7 genes, including *gcm* and *gcm2*, were also tested. In all of these cases, we saw the same ISNb defects as in Df(2L)Exel7042, with similar penetrances (data not shown).

To examine how the absence of glia affects the ISNb, we stained Df(2L)Exel7042 embryos with an antibody to Fasciclin III (FasIII), which is a marker for the cell bodies and proximal axons of the ISNb neurons RP1, RP3, RP4 and RP5 [28]. In wild-type embryos, FasIII staining reveals a regular pattern in which RP axons cross the midline and then extend posteriorly for a short distance before entering the ISN root (Figure 5D). In Df(2L)Exel7042 embryos, a variety of defects were seen that could account for the different classes of guidance errors we see in ISNb (Figure 5E). We found that in many segments of the embryos extra cells express FasIII. This is likely due to the fate switches from glial to neuronal that occur in the absence of *gcm* and *gcm2* function. We also observe that in some segments neurons of the RP cluster fail to extend axons. In other segments they extend axons that enter the longitudinal tracts, but then exit the CNS in the wrong place or never exit at all. In some cases the axons grow anteriorly rather than posteriorly along the longitudinal tracts. All of these phenotypes could result in the loss of the ISNb in the periphery, as well as contributing to the observed breaks in the longitudinal tracts.

Are the defects we see in Df(2L)Exel7042 due to a lack of glial cells, or to loss of some other function of *gcm* and *gcm2*? In order to test whether glia are required for ISNb axon guidance we examined *reversed polarity* (*repo*) mutants. *repo* encodes a transcription factor that is downstream of *gcm* and is also required for glial cell fate [21], [29], [30], [31]. In the absence of Repo, few glial cells differentiate. We stained embryos homozygous for a null allele of *repo* with 1D4. We found that the longitudinal axon tracts in *repo* embryos are wavy and have breaks (Figure 5C). There are extra midline cells expressing FasIII in some segments. We also observed guidance errors with anti-FasIII in which RP axons turn anteriorly along the longitudinal pathway. However, these defects are not as penetrant as when *gcm* and *gcm2* are both deleted (Figure 5F). In the periphery, the ISNb is usually normal in *repo* embryos, although there are rare stall phenotypes (Figure 5I). This result might be explained by the fact that although *repo* is required for normal glial cell fate, in the absence of *repo* glia still begin to differentiate and express some glial antigens [29], [30], [31].

### Dfs causing changes in antigen expression

**Figure 6:**
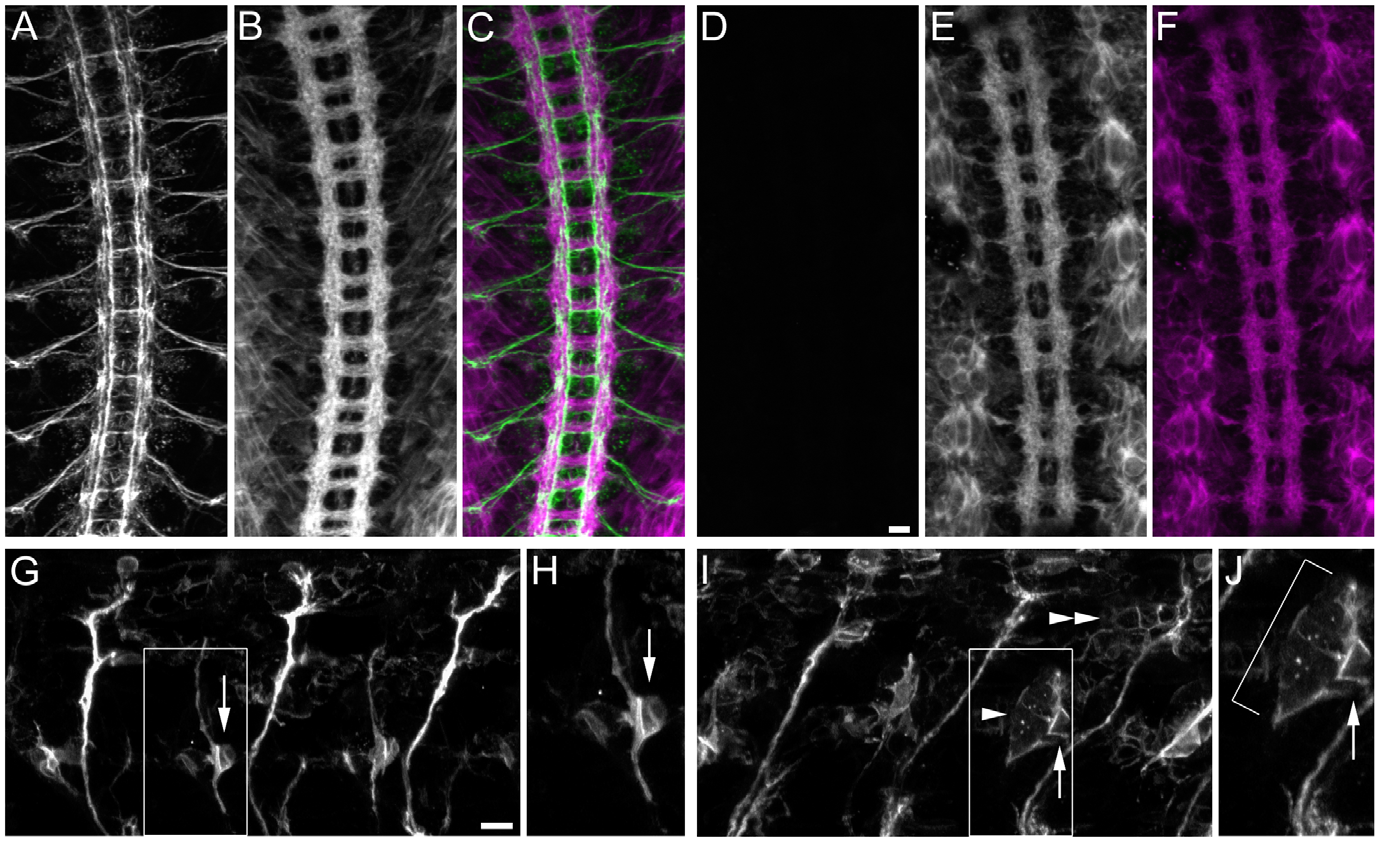
Deficiencies causing loss or ectopic expression of 1D4 antigen. Panels A–F are maximum intensity confocal projections of stage 16 live-dissected embryos double-stained with mAb 1D4 and rhodamine-phalloidin (20× objective). Anterior is up. Scale bar equals 10 µm. (A) 1D4 staining in a wild-type embryo. (B) Phalloidin staining in a wild-type embryo. (C) Wild-type merged image (1D4, green; phalloidin, magenta). (D) 1D4 staining in a Df(1)C128 embryo. The image is black because these embryos completely lack 1D4 antigen. (E)Phalloidin staining in a Df(1)C128 embryo, showing than CNS axons are present. (F) Df(1)C128 embryo merged image. Panels G and I are confocal maximum intensity projections of stage 16 live-dissected embryos stained with 1D4 (63× objective). Anterior is to the left and dorsal is up. Scale bar equals 7 µm. Panels H and J are equivalent to the boxed areas in G and I. (G) 1D4 staining in 3 abdominal hemisegments in a wild-type embryo. FasII expression is mostly restricted to neurons, although there is some weak staining of other cell types. The LBD neuron is indicated by an arrow. (H) Detail of boxed area in G. The LBD is indicated by an arrow. (I) 1D4 staining in 3 abdominal hemisegments in a Df(3R)Exel7310 embryo. Bright FasII expression is seen on non-neuronal cells, including flat cells (arrowhead) adjacent to the LBD (arrow). Other 1D4-expressing cells are located more dorsally, just anterior to the ISN (double arrowhead). (J) Detail of boxed area in (I). The LBD is indicated by an arrow. The bracket indicates the group of cells that ectopically express 1D4 antigen.

Another potential use of Df screening would be to find genes required for normal cellular or subcellular expression of proteins that can be visualized by antibody staining. In the course of our examination of Df homozygotes by 1D4 staining, we found two Dfs for which homozygotes (or hemizygotes) lack 1D4 antigen. One of these, Df(1)C128, is shown here (Figure 6D). Df(1)C128 embryos clearly contain CNS axons, as shown by rhodamine-phalloidin staining (Figure 6E), and they also stain with BP102 (data not shown). Df(1)C128 does not delete the FasII gene, and we confirmed that the line does not harbor a *FasII* mutation by complementation testing. The most likely explanation for the loss of FasII expression is perhaps that Df(1)C128 deletes a gene encoding a transcription factor necessary for production of *FasII* mRNA. Embryos homozygous for Df(3R)Exel7310 display 1D4 staining on cells in the periphery that normally do not exhibit staining. These are large, flat cells just anterior to the LBD neuron (Figures 6I, J). We do not know the identities of these cells. They appear to be internal to the epithelial layer, and they do not have morphologies like those of muscles or sensory neurons. Two possible explanations for this the presence of this ectopic 1D4 staining are: 1) the Df deletes a gene encoding a repressor that prevents the FasII gene from being transcribed in these cells; 2) it removes a gene whose product normally cleaves FasII off the surfaces of these cells. Other models are also possible.

## Discussion

In this paper, we define new kits of publicly available Dfs that can be used for a variety of phenotypic screens. A set of ∼400 Dfs (the ‘complete kit’) for which homozygotes develop well enough to have a recognizable CNS and body walls encompasses 82% of polytene chromosome bands (Table S1). This kit can be screened for orphan receptor ligands (Figure 1) and antigen expression (Figure 6). A subset of the complete kit, covering ∼50% of polytene chromosome bands, contains 233 lines with ‘mild’ phenotypes, for which body walls have a wild-type morphology and the CNS has a relatively normal structure (Table S2). This subset kit can be used to screen for regions of the genome whose deletion confers specific embryonic phenotypes. We screened it for axon guidance defects, and identified many new regions containing genes necessary for establishment of motor axon and CNS pathways (Figures 2–5).

### Development, characterization, and use of the ‘complete’ Df kit

Our initial screen for orphan receptor ligands was performed with the Bloomington Df kit as it existed in 2002 [11]. This kit contained a large number of Dfs for which homozygotes failed to develop, and most of the Dfs were only defined at cytological resolution. To create a more useful kit for phenotypic screening, we analyzed hundreds of additional Dfs for phenotype and ligand staining, so as to define the smallest possible collection of Dfs that would allow genome-wide ligand and phenotype screening.

In 2009, Kevin Cook and his colleagues at the Bloomington stock center replaced the old Bloomington kit with a new kit of 467 molecularly defined deletions [32], [33]. They were not interested in phenotype when they created this kit, but only in maximum coverage by a minimum number of Dfs. Our project was carried out independently of their work, screening new Dfs for phenotype and adding them to our kit as they were generated by the community. Thus, although our kit is almost entirely composed of lines available from Bloomington, only 31% of the lines in our kit are in the new Bloomington Df mapping kit. Since we retained Dfs from the old Bloomington kit for which homozygote embryos developed well, our complete kit contains 185 lines for which the Dfs are still not molecularly mapped. In the past, this would have made gene localization under the Dfs difficult. Now, however, because so many molecularly mapped Dfs are available, if one finds a ‘hit’ with a Df mapped only to cytological resolution, overlapping molecularly mapped Dfs can immediately be obtained and screened in order to localize the relevant gene to a molecularly defined interval.

To screen the genome for orphan receptor ligands, the first step is to generate fusions of the XC domain of the receptor of interest to AP, an obligate dimer [13]. These dimeric fusion proteins are expressed at high levels using the baculovirus system, and unpurified supernatants are used directly for staining. Live-dissected embryos of various ages are incubated with fusion protein supernatants, followed by fixation, anti-AP secondary antibody, and tertiary fluorescent antibody. Once robust staining of a potential ligand(s) has been obtained, the Df collection is screened by double-staining with receptor fusion protein and a monoclonal antibody that recognizes the tissue in which the ligand is expressed. This is necessary in order to ensure that the absence of staining in homozygotes for a particular Df is not due to the absence of the relevant tissue. Some advantages of this method over other ways of identifying orphan receptor ligands, such as cDNA expression cloning in mammalian cell systems, include: 1) since it is a search for genes that are necessary for ligand expression, not genes sufficient for expression, it can identify components of multimeric ligands, which could not be found by expression cloning; 2) its success does not depend on the abundance of the mRNA encoding the ligand(s); and 3) the screen identifies not only the ligand genes themselves, but other genes required for ligand expression or localization. For example, in the screen that identified Syndecan, we also found genes encoding modification enzymes necessary for HSPG synthesis [11].

This same type of screen can also identify genes required for expression or localization of any antigen recognized by a high-quality antibody. In our screen, we found Dfs that eliminated expression of the FasII protein. We also found Dfs that caused FasII to be expressed in different cell types than in wild-type embryos (Figure 6). The responsible genes under these Dfs might encode transcription factors necessary for *FasII* mRNA expression, or proteins required for transport of FasII to the cell surface.

The complete kit could also facilitate identification of genes required for appropriate subcellular localization of a protein. For example, if one screened with an antibody recognizing an apical protein, one could find genes necessary for its localization to the apical side of the cell. One could also screen for genes necessary for delivery of proteins to axons or dendrites. Kit screening could also be done using fluorescent *in situ* hybridization. This would allow identification of genes required for appropriate expression of a transcript, even if one does not have a good antibody against the protein encoded by the transcript.

### Using subset kits to screen for phenotypes

We systematically examined the subset kit of ‘mild’ Df lines for motor axon phenotypes, and defined 20 Dfs that cause SNa bifurcation defects, 9 that produce ISNb defects, and others that cause ISN and SNc defects. At least 24 produce muscle phenotypes (Table 1, Table S3). We also found many lines with specific CNS axon defects (Figure 2).

The identification of 9 regions selectively affecting ISNb guidance within the ∼40% of the genome covered by the mild Dfs we screened suggests that there might be a total of ∼22 genes with ISNb-specific loss-of-function (LOF) phenotypes in the whole genome. This seems like a surprisingly small number of genes, but it is important to note that single mutations in many axon guidance genes produce only low-penetrance defects. Deletion of such genes might produce no phenotype, or a phenotype whose penetrance would fall below the threshold (∼20%) required for us to classify the Df as abnormal. For example, loss of either Ptp69D or Ptp99A produces weak ISNb phenotypes, but a double mutant has strong phenotypes [34], [35]. *robo2* (*leak*) mutants have weak CNS phenotypes, but *robo2* mutations strongly enhance the *robo* phenotype, so that the *robo robo2* double mutant phenotype resembles that of *slit* [36]. Thus, because of genetic redundancy, there are probably many genes involved in axon guidance that cannot be discovered by LOF screens. Such genes must be identified by candidate gene approaches, like those used to find *Ptp69D*, *Ptp99A*, and *robo2*, or by conducting enhancer/suppressor screens.

The problem of genetic redundancy can be sometimes be addressed using Dfs, since closely related genes are often located near each other. One of the regions we identified as affecting ISNb guidance contains *gcm* and *gcm2*, which encode transcription factors that contribute to glial cell fate determination. When both *gcm* and *gcm2* are deleted, there are no glia at all, and development of the RP cell bodies and axon tracts is abnormal (Figure 5). In some segments, the ISNb is completely absent because no RP axons leave the CNS. In others, the ISNb appears to stall, possibly due to the absence of the RP axons that normally innervate muscles 12 and 13. ISNb bypass phenotypes could be due to the absence of glial cells near the exit junction that are required for normal separation of the ISNb and ISN axons. These ISNb phenotypes were not found before because embryos lacking both Gcm proteins had never been examined for motor axon defects. They are almost never observed when only *gcm* is mutant, perhaps because a few glia are still present in these mutants due to Gcm2 expression.

The use of Dfs allowed us to uncover the roles of Gcm proteins in motor axon guidance. However, in some cases Df phenotypes are observed that are not due to loss of a single gene, and cannot be so easily assigned to loss of a cluster of related genes. In such instances, it could be difficult to identify the specific combination of genes whose loss causes the phenotype.

The use of the Df kit has also allowed us to discover phenotypes that had not been seen previously. These include the looped ISNb phenotype (Figure 4), SNc-specific phenotypes, and new phenotypes affecting multiple motor axon pathways. Since most tissues appear to develop normally in homozygotes for mild Dfs, this kit should provide a valuable resource for other groups that are interested in screening for new phenotypes affecting any embryonic organ. The only requirement for performance of such a screen is a good antibody that recognizes structures within the tissue in question. Such screens can systematically survey ∼50% of the genome for any phenotype that can be visualized at high magnification under a compound or confocal microscope. A single person could conduct such a screen in a period of months, making it a much less daunting project than the execution of an EMS or *P* element screen.

## Materials and Methods

### Genetics

Most Df strains and mutants were obtained from the Bloomington Stock Center. A few Df lines were obtained from the Szeged and Kyoto Stock Centers. Deficiency kit lines were balanced over FM7c, P(Gal4-Kr.C)DC1, P(UAS-GFP.S65T)DC5, sn-, CyOarmGFP, TM3armGFP, CyO-Wingless-LacZ, or Tm6B-Ubx-LacZ (Bloomington).

### Overview of screening procedures

For identification of homozygote embryos, Dfs are placed over GFP balancer chromosomes, so that Df/Df embryos can be recognized prior to dissection. 29 Dfs on the X, 2^nd^, and 3^rd^ chromosomes cannot be maintained over these balancers and must be screened blind. There are also 9 4^th^ chromosome Dfs that lack GFP balancers.

To screen the kit for ligand or antigen expression, one collects embryos from groups of up to 10 lines at a time, sorts them for GFP expression, lines up GFP-minus embryos in rows on a glass slide, and dissects 4 or 5 embryos for each line. If the Dfs are being screened for antigen expression or phenotype, they are immediately fixed and stained with the appropriate antibody. If a ligand screen is being conducted, the embryos are first incubated live with receptor fusion proteins, followed by fixation and detection of fusion protein binding with secondary and tertiary antibodies [11]. To facilitate screening, we have published a detailed video protocol for sorting, live dissection, and staining of embryos [12].

### Immunohistochemistry/immunofluorescence and microscopy

GFP fluorescence from the balancer chromosome was scored using an Olympus GFP dissecting microscope. For 1D4 fluorescent staining, embryos are dissected live on glass slides, fixed and washed in PBS. Embryos are then washed in PBS +0.5% Triton (PBT), blocked with PBT +5% heat-inactivated normal goat serum (NGS) and incubated overnight in a dilution of 1∶3 1D4 in PBT +5% NGS. After washing in PBT embryos are incubated at 4C overnight in AlexaFluor anti-mouse 488 secondary antibody and rhodamine-phalloidin (Invitrogen). For fusion protein staining, embryos are incubated in fusion protein for 2 hours after dissection in the absence of detergent. Samples are fixed, washed, and incubated in rabbit-anti-AP and BP102 overnight at 4°C and then processed with appropriate secondary antibodies. Samples are mounted in 70% glycerol in PBS.

Fixed embryo immunohistochemistry was performed as previously described [3]. Briefly, after fixation embryos are extensively washed in PBS +0.5% Triton (PBT), incubated overnight at 4°C in PBT +5% heat-inactivated goat serum (HIGS) with a 1∶5 dilution of 1D4 antibody plus 1∶5000 dilution of rabbit-anti-beta-galactosidase antibody (MP Biomedicals) to label embryos carrying the balancer chromosomes. Embryos are extensively washed in PBT and then incubated overnight at 4°C in PBT +5% HIGS with 1∶500 dilutions of horseradish peroxidase-conjugated goat-anti-mouse and goat anti-rabbit (Jackson Immunoresearch). After extensive washing in PBT, the peroxidase reaction was carried out using a DAB peroxidase substrate kit (Vector Laboratories), and the embryos were mounted in 70% glycerol and dissected.

Embryos were scored for fusion protein staining, motor axon defects, CNS defects, and muscle defects on a Zeiss Axioplan microscope with a 40× multi-immersion objective. Screening for CNS, motor axon, and muscle defects was conducted by scoring 5 homozygous deficiency embryos per stock. Abdominal hemisegments A2–A7 were scored. Quantitative data was obtained by analyzing 1D4 HRP immunoreactivity. Homozygous deficiency embryos were scored blind for axon guidance defects in abdominal hemisegments A3–A7.

The following antibodies were used rabbit anti-AP (Serotec) 1∶600, mAb bp102 1∶30, mAb 1D4 1∶3, mAb7G10 1∶5 (Caltech Monoclonal Antibody Facility), AlexaFluor anti-mouse 488, Alexa Fluor anti-mouse 568, and AlexaFluor anti-rabbit 488 (Invitrogen) 1∶1000. Horseradish peroxidase-conjugated goat-anti-mouse and goat anti-rabbit (Jackson Immunoresearch) 1∶500. Rhodamine-phalloidin (Invitrogen) was used at 1∶2000 to detect muscle structure.

Confocal imaging was performed using a Zeiss LSM inverted microscope using 20×, 40×, and 63× Zeiss oil-immersion objectives. Stacks were projected using Image J software maximum intensity projections.

## Supporting Information

Table S1 Headings for columns A-D are self-evident or are explained in the paper text. For the other columns, ‘m’ (column E) refers to whether a Df is molecularly mapped. ‘o’ (column F) is the suggested order of kit screening, where (1) is the collection of Dfs that are mild, moderate, or severe and are balanceable over GFP, (2) is the collection of Dfs that are balanceable over GFP but may be redundant with (1), (3) are Dfs that can't be balanced over GFP, and (4) are very severe Dfs that are only marginally screenable. Mild Dfs are those which have a normal BP102 ladder and normal overall body wall structure; moderate Dfs are those which have some visible abnormalities in the CNS axon ladder; severe Dfs are those which have a disorganized CNS but still contain large numbers of axons; very severe Dfs are those which have highly disorganized CNS structure usually with few axons and overall structure of the embryo is abnormal. Annotation of lines as DK1, DK2, and DK3 refers to the old (2002) Df kit from the Bloomington Stock Center, not the new molecularly mapped kit. ‘Notes on lines’ (column G) contains useful information about the Dfs and the reasons for their incorporation into the kit; it also contains instructions for screening nonbalanceable Df lines, and notes on regions covered only by Dfs that cannot be screened. ‘Further notes on mapping’ (column H) contains complementation data and overlap information from the old Bloomington kit.
(0.10 MB XLS)

Table S2 The ‘mild’ subset of the deficiency kit
(0.05 MB XLS)

Table S3 Headings for columns A and B are self-evident. ‘Notes’ (Column C) contains information about the phenotype of the Df such as the affected motor pathway, muscle defect, or Dfs which are developmentally delayed.
(0.03 MB XLS)
